# Acute sleep deprivation enhances susceptibility to the migraine substrate cortical spreading depolarization

**DOI:** 10.1186/s10194-020-01155-w

**Published:** 2020-07-06

**Authors:** Andrea Negro, Jessica L. Seidel, Thijs Houben, Esther S. Yu, Ike Rosen, Andrea J. Arreguin, Nilufer Yalcin, Lea Shorser-Gentile, Lea Pearlman, Homa Sadhegian, Ramalingam Vetrivelan, Nancy L. Chamberlin, Cenk Ayata, Paolo Martelletti, Michael A. Moskowitz, Katharina Eikermann-Haerter

**Affiliations:** 1 Neurovascular Research Laboratory, Department of Radiology, Massachusetts General Hospital, Harvard Medical School, Charlestown, MA USA; 2 Department of Clinical and Molecular Medicine, Sapienza University of Rome, Rome, Italy; 3 Department of Neurology, Beth Israel Deaconess Medical Center, Harvard Medical School, Boston, MA USA; 4 Department of Pathology, Boston Children’s Hospital, Boston, MA USA; 5 Stroke Service and Neuroscience Intensive Care Unit, Department of Neurology, Massachusetts General Hospital, Harvard Medical School, Boston, MA USA; 6 Department of Radiology, and Department of Neurology, Massachusetts General Hospital, Harvard Medical School, Boston, MA USA; 7 Department of Radiology, Massachusetts General Hospital, Harvard Medical School, 55 Fruit Street, Boston, MA 02114 USA

**Keywords:** Cortical spreading depolarization, CSD, Migraine, Sleep deprivation, Ventrolateral preoptic (VLPO) nucleus, VLPO

## Abstract

**Background:**

Migraine is a common headache disorder, with cortical spreading depolarization (CSD) considered as the underlying electrophysiological event. CSD is a slowly propagating wave of neuronal and glial depolarization. Sleep disorders are well known risk factors for migraine chronification, and changes in wake-sleep pattern such as sleep deprivation are common migraine triggers. The underlying mechanisms are unknown. As a step towards developing an animal model to study this, we test whether sleep deprivation, a modifiable migraine trigger, enhances CSD susceptibility in rodent models.

**Methods:**

*Acute* sleep deprivation was achieved using the “gentle handling method”, chosen to minimize stress and avoid confounding bias. Sleep deprivation was started with onset of light (diurnal lighting conditions), and assessment of CSD was performed at the end of a 6 h or 12 h sleep deprivation period. The effect of *chronic* sleep deprivation on CSD was assessed 6 weeks or 12 weeks after lesioning of the hypothalamic ventrolateral preoptic nucleus. All experiments were done in a blinded fashion with respect to sleep status. During 60 min of continuous topical KCl application, we assessed the total number of CSDs, the direct current shift amplitude and duration of the first CSD, the average and cumulative duration of all CSDs, propagation speed, and electrical CSD threshold.

**Results:**

*Acute* sleep deprivation of 6 h (*n* = 17) or 12 h (*n* = 11) duration significantly increased CSD frequency compared to controls (17 ± 4 and 18 ± 2, respectively, vs. 14 ± 2 CSDs/hour in controls; *p* = 0.003 for both), whereas other electrophysiological properties of CSD were unchanged. *Acute* total sleep deprivation over 12 h but not over 6 h reduced the electrical threshold of CSD compared to controls (*p* = 0.037 and *p* = 0.095, respectively). *Chronic* partial sleep deprivation in contrast did not affect CSD susceptibility in rats.

**Conclusions:**

*Acute* but not *chronic* sleep deprivation enhances CSD susceptibility in rodents, possibly underlying its negative impact as a migraine trigger and exacerbating factor. Our findings underscore the importance of CSD as a therapeutic target in migraine and suggest that headache management should identify and treat associated sleep disorders.

## Introduction

Migraine is a multifactorial neurovascular disorder characterized by recurrent episodes of headache. One third of patients experience transient neurological symptoms associated with their attacks, the so-called migraine aura. Migraine is among the most common neurological diseases, affecting approximately 20% of the adult population [1]. The socioeconomic impact is high [2, 3], and the World Health Organization recognized migraine as a major public health problem by ranking it as #2 among all diseases causing disability [4] and as #1 in under-50-year-old humans of both genders [5]. Migraine attacks may increase in frequency over time, and approximately 2.5% of patients with episodic migraine develop chronic migraine [6]. The transition to more frequent attack patterns is influenced by genetic predisposition, co-morbid conditions, life events and lifestyle [7].

Sleep disorders are well known risk factors for migraine chronification [7, 8] and changes in wake-sleep patterns such as sleep deprivation are common migraine triggers [9]. Compared to a few decades ago, adults sleep less, and sleeping as little as possible is often seen as an admirable behavior. Sleep loss may result from total sleep deprivation (such as shift workers might experience), chronic sleep restriction (due to work, medical conditions or lifestyle), or sleep disruption (in sleep disorders such as sleep apnea or restless legs syndrome) [10]. Sleep disorders are frequently observed in specific headache diagnoses (e.g., migraine, tension-type headache, cluster headache) [11] and other nonspecific headache patterns (i.e., chronic daily headache, hypnic headache, “awakening” or morning headache) [12]. In migraineurs, sleep disorders are well known predictive factors of progression from episodic to chronic forms [13]. In contrast, a targeted behavioral sleep intervention can lead to an improvement in headache frequency and reversion from chronic to episodic migraine [14]. The sleep deprivation-induced increase in adenosine levels might be an underlying mechanism, because adenosine levels are elevated during migraine attacks, and administration of adenosine can precipitate migraine attacks [15].

Cortical spreading depolarization (CSD), discovered by Leão in 1944 [16], is considered the electrophysiological correlate of migraine aura, and a possible trigger of migraine headache [17]. CSD is an intense propagating depolarization of neuronal and glial membranes. Evoked when the local extracellular potassium concentration [K^+^]_e_ exceeds a critical threshold, CSD causes a loss of membrane resistance and massive ionic disturbances with cell swelling. Genetic and environmental factors important for the clinical manifestation of migraine have been shown to modulate cortical excitability, including the potential to elevate [K^+^]_e_ and glutamate to levels sufficient to initiate and facilitate CSD. For example, mutations causing the migraine-associated syndromes familial hemiplegic migraine (FHM) or cerebral autosomal dominant arteriopathy with subcortical infarcts and leukoencephalopathy (CADASIL) enhance CSD susceptibility [18, 19]. Similarly, gonadal hormones modulate CSD susceptibility, to explain the female preponderance in migraine [18, 20]. In the setting of sleep deprivation, adenosine overload with overstimulation of A1 receptors is a possible mechanism modulating neuronal activity and CSD, because adenosine A_1_ receptor activation contributes to the persistent secondary phase of Leão’s cortical spreading depression [21, 22].

The current study found that *acute* sleep deprivation as a modifiable environmental factor enhances CSD susceptibility, possibly explaining its debilitating effects on migraine.

## Materials and methods

### Experimental groups

All experimental procedures were carried out in accordance with the Guide for Care and Use of Laboratory Animals (NIH Publication No. 85–23, 1996), and were approved by the institutional review board (MGH Subcommittee on Research Animal Care, SRAC) and Institutional Animal Use and Care Committee of Beth Israel Deaconess Medical Center. All animals were housed individually under controlled conditions (12 h of light starting at 08:00) in an isolated ventilated chamber maintained at 20–22 °C and given access to food and water ad libitum. Only male rodents were used in this study to reduce the number of animals needed to control for hormonal fluctuations during the female estrus cycle [23]. CSD recordings were carried out by an investigator blinded to the sleep deprivation status.

- ***Acute Sleep Deprivation:***

A total of 58 rats (Sprague-Dawley, male, 340 ± 40 g; Charles River Laboratories, Wilmington, MA) were used to test the hypothesis that acute sleep deprivation enhances CSD susceptibility. Experimental groups consisted of 30 control rats as well as 11 rats after 12 h of sleep deprivation and 17 rats after 6 h of sleep deprivation. From the group of 30 control rats, we used 11 rats as controls interleaved with rats after 6 h of sleep deprivation and 19 rats as controls interleaved with rats after 12 h of sleep deprivation. Acute sleep deprivation was induced using the “gentle handling method” [24], chosen to minimize stress and avoid any confounding bias associated with enforced waking present with other techniques (e.g. lights, sounds, water tank). For the same reason, the “gentle handling method” was preferred over alternative techniques that use stimuli known to also serve as migraine triggers, e.g., stress, pain, light, smell or sound [25], which could have introduced additional confounding bias. Rats were kept awake by providing new objects into their home cage when they adopted a sleeping posture, i.e., curled up with eyes closed. Sleep deprivation was started with the onset of light cycle (diurnal lighting conditions, 8 am - 8 pm), and assessment of CSD was performed at the end of the 6 h or 12 h sleep deprivation period. Control rats were kept in the same environment but left undisturbed. Sleep-deprived rats and control rats were housed individually, in the same room. Every day, one control rat and one sleep-deprived rat were randomized and coded with the letter “A” or “B” on their cage card, by a person not involved in CSD recordings. The investigator performed 2 CSD recordings per day, testing 1 control rat and 1 sleep-deprived rat, in random order. The investigator pulled a card with “A” or “B” before CSD recordings, and started CSD recordings with the respective rat accordingly, not knowing the rat’s sleep deprivation status.

b) ***Chronic sleep deprivation***

A total of 29 rats (Sprague-Dawley, male, 405 ± 30 g; Taconic, Germantown, NY) were used to test the effect of partial chronic sleep deprivation on CSD susceptibility. To minimize stress and avoid any confounding bias associated with enforced waking, chronic sleep deprivation was induced by cytotoxic lesioning of the hypothalamic sleep-promoting cell group, the ventrolateral preoptic (VLPO) nucleus [26]. All rats were implanted with electrodes for recording electroencephalogram (EEG) and electromyogram (EMG) for assessing their sleep-wake status. A prelesion control EEG of the rats was continuously recorded for 24 h (13:00–13:00), 6 days after the EEG implantation. Wake–sleep states were manually scored, by an observer who was not aware of the histological results of the animals, in 12 s epochs based on the digitized EEG of each rat. Wakefulness was identified by the presence of desynchronized-EEG.

Animals were anesthetized with chloral hydrate and positioned in a stereotaxic frame. The skull was reexposed, and the tip of a fine glass micropipette was placed at the VLPO stereotaxic coordinates (AP − 0.6 from bregma, L ± 1.00, DV 8.5 as per the rat atlas of Paxinos and Watson). Two hundred nanoliters of 0.1% orexin-B-saporin (Advanced Targeting Systems, CA, USA) or saline as a control were injected by air pressure and the pipette was slowly removed after 2 min. In this manner, each rat received bilateral injections targeted at the VLPO. Two weeks after the surgical procedure, rats were habituated to the recording conditions and sleep-wake recordings (EEG/EMG) were performed for 24 h. In order to assess the cumulative effect of chronic sleep deprivation, CSD recordings (as described below) were performed 6 weeks or 12 weeks after VLPO lesions. Following CSD characterization, rats were perfused with saline (100 ml) followed by 10% formalin (300 ml) transcardially and their brains were cut in the coronal plane on a freezing microtome into four evenly spaced series of 40 μm coronal sections and Nissl stained as described previously [26, 27]. VLPO lesioning was considered successful in cases of > 70% VLPO neuron loss bilaterally, which has been shown repeatedly in multiple studies to cause a 50% decrease in NREM sleep time [26]. The control group consists of rats with off-target lesions, and sham-lesioned (saline-injected) rats.

### Surgical preparation for CSD assessment

**Fig. 1:**
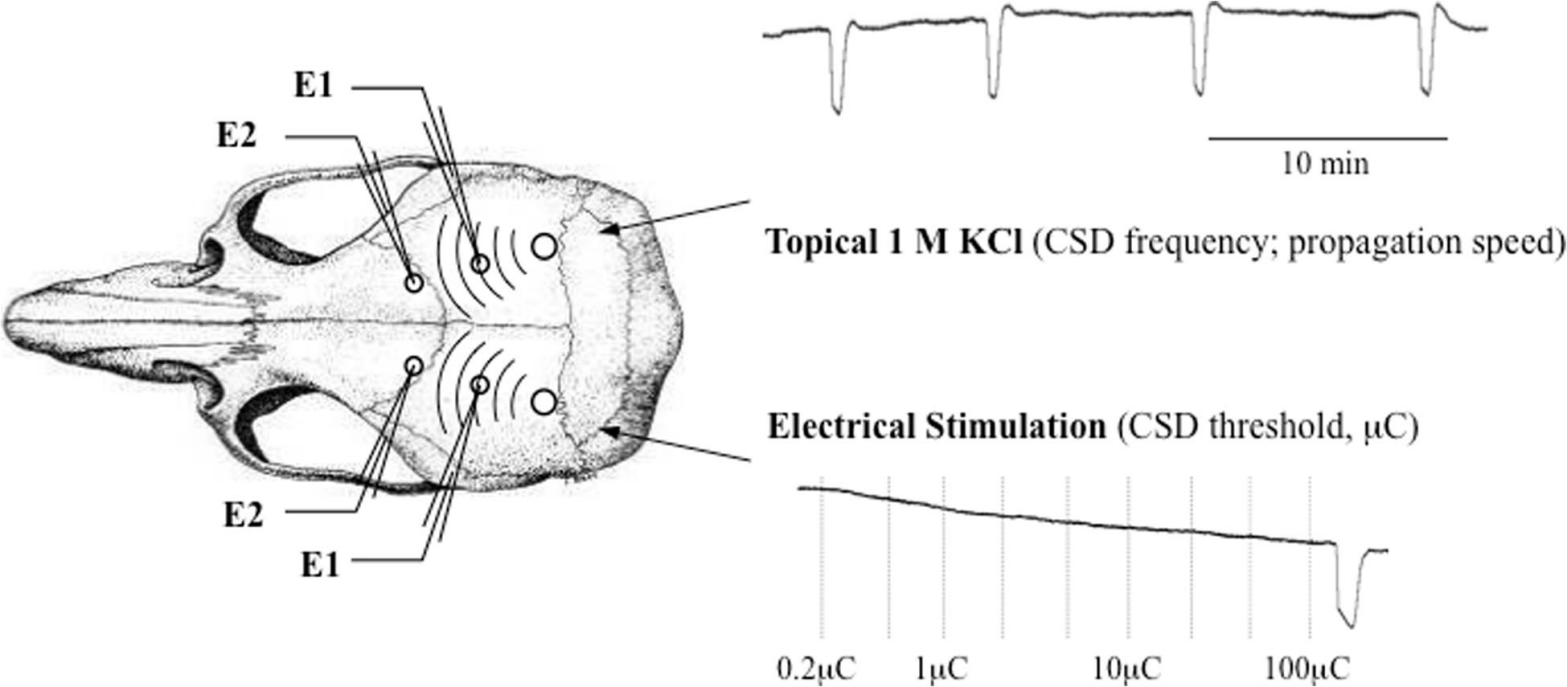
Experimental setup for electrophysiological recordings. CSD, cortical spreading depolarization; E, electrode; μC, micro coulomb

Rodents were anesthetized with isoflurane (2.5% induction, 1% maintenance, in 70% N_2_O / 30% O_2_), and tracheostomized for mechanical ventilation (SAR-830, CWE, Ardmore, PA, USA). A femoral artery was cannulated for continuous mean arterial blood pressure recording (PowerLab; AD Instruments, Colorado Springs, CO, USA). Arterial blood gas and pH were maintained within normal limits by adjusting the ventilation parameters (Rapidlab 248 blood gas/pH analyzer, Siemens Healthcare Diagnostics, Tarrytown, NY, USA). Rectal temperature was maintained at 36–37 degrees Celsius using a thermostatically controlled heating pad (FHC, Bowdoinham, ME, USA). Rodents were placed in a stereotaxic frame (Stoelting, Wood Dale, IL, USA) and 3 burr holes were drilled bilaterally for electrophysiological recordings (recording site 2), as described previously (Fig. 1) [28].

### Electrophysiological CSD recordings

All experiments were performed in a blinded fashion with respect to sleep status. Two glass capillary microelectrodes were placed to measure the potential difference between recording sites 1 and 2 on each side of the brain to record extracellular steady (DC) potential changes and electrocorticogram (FHC, Inc. Bowdoinham, ME), using a data acquisition system for off-line analysis (PowerLab; AD Instruments, Colorado Springs, MO, USA). The electrical stimulation threshold for CSD was assessed by direct cortical stimulation with a bipolar stimulation electrode placed on the occipital cortex (400 μm tip diameter, 1 mm tip separation; Frederick Haer Company, Bowdoin, ME, USA). Using a stimulator (GrassInstruments, West Warwick, RI, USA) and a constant current unit (WPI, Sarasota, FL, USA), cathodal squared pulses of increasing intensity and duration (1–4000 μC) were applied at 4 min intervals, until CSD occurred. For KCl-induced CSD susceptibility, a 1.5 mm cotton ball soaked with 1 M KCl was placed on the pial surface of the contralateral hemisphere and replaced every 15 min. The total number of CSDs during 60 min of continuous topical KCl application was counted. The DC shift amplitude and duration (sec) (measured at half maximal amplitude) of the first CSD, as well as the propagation speed (mm/min), the propagation failure (%) and the cumulative duration (sec) of all CSDs during continuous KCl application were calculated.

### Statistics

Data were analyzed with SPSS (version 11.0). Using a general linear model of covariance analysis (ANACOVA), we tested for an effect of the independent variable sleep deprivation on the dependent variables CSD threshold, frequency and propagation speed. Other electrophysiological measures of CSD and systemic physiological data were compared among groups using one-way ANOVA. Data are shown as mean ± standard deviation, and *p* < 0.05 was considered significant.

## Results

Mean arterial blood pressure, arterial pCO_2_ and pH were within normal physiological range and did not differ among groups (Table 1).

**Table 1:** Physiological parameters

**Group**	**pH**	**paCO2**	**paO2**	**BP**
Control	7.41 ± 0.03	42 ± 2	117 ± 18	83 ± 9
6 h sleep deprivation	7.41 ± 0.02	40 ± 3	123 ± 28	92 ± 11
12 h sleep deprivation	7.41 ± 0.03	39 ± 2	119 ± 22	89 ± 8
**Group**	**pH**	**paCO2**	**paO2**	**BP**
VLPO-lesioned	7.42 ± 0.04	39 ± 6	132 ± 21	99 ± 12
Control	7.39 ± 0.05	41 ± 8	118 ± 37	90 ± 17

Physiological parameters did not differ between groups. A) Acute sleep deprivation in rats: 6 h controls and 12 h controls were pooled for statistical analysis, as no significant difference was found between the two control groups; B) Chronic sleep deprivation in VLPO-lesioned rats. Control animals underwent a sham procedure with saline injection into their VLPO

The arterial blood gas (mmHg) and pH values were measured at the beginning and end of each KCl stimulation period, and before electrical stimulation, for a total of 6 measurements per experiment. Data are presented as average ± SD. Arterial blood pressure (BP) values (mmHg) are the average of the KCl application and electrical stimulation recording periods

### Acute sleep deprivation increased CSD susceptibility

**Fig. 2:**
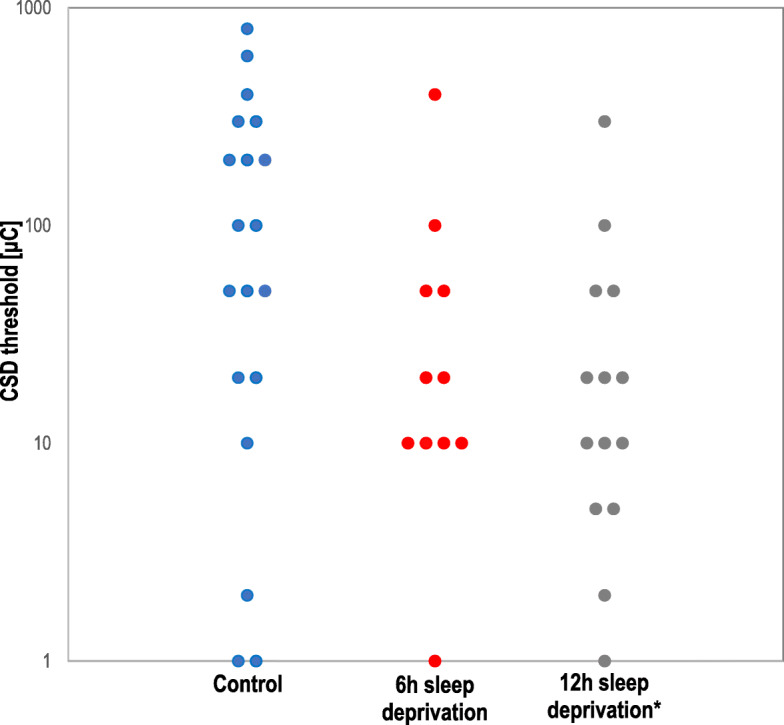
Effect of acute sleep deprivation on electrical CSD threshold. CSD threshold was reduced after 12 h but not 6 h of sleep deprivation. Each circle represents the CSD threshold of an individual rat. All controls (6 h controls and 12 h controls) were pooled for statistical analysis, as no significant difference was found between the two control groups. * *p* < 0.01 vs. control

**Fig. 3:**
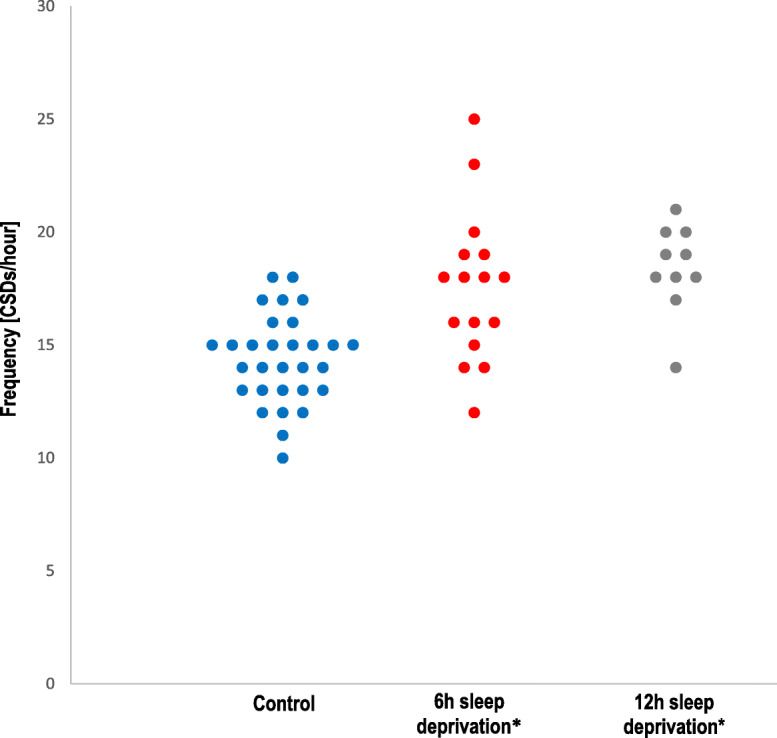
Effect of acute sleep deprivation on CSD frequency. Both 6 h and 12 h of sleep deprivation increased the frequency of CSD during topical continuous application of KCl. Each circle represents the CSD frequency of an individual rat. All controls (6 h controls and 12 h controls) were pooled for statistical analysis, as no significant difference was found between the two control groups. * *p* = 0.03 vs. control

Twelve hours but not 6 h sleep deprivation significantly reduced the electrical threshold for CSD induction, when compared to controls (*p* = 0.037 and *p* = 0.095, respectively; Fig. 2). Data from 6 h and 12 h control animals were pooled as there were no differences for any study endpoint. Overall, sleep deprived rats showed a reduced electrical threshold of CSD compared to controls (*p* = 0.015) (Table 2). Continuous application of 1 M KCl on the occipital cortex evoked repetitive CSDs in all groups. Control rats (*n* = 30; 6 h and 12 h) developed 14.4 ± 2.0 CSDs/hour. Both 6 h (*n* = 17) and 12 h (*n* = 11) sleep-deprivation increased CSD frequency compared to respective-interleaved controls (16.9 ± 3.5 and 17.9 ± 2.1, respectively; *p* = 0.003 for both; Fig. 3), while other electrophysiological properties of CSD were unchanged. Recordings from 6 h or 12 h sleep-deprived rats and controls did not differ for propagation speed (4.6 ± 0.7, 4.5 ± 0.5 and 4.2 ± 0.6) and propagation failure (31.9 ± 13.2, 30.0 ± 11.8 and 31.5 ± 11.8) (Table 3), neither for the duration of first CSD (23.4 ± 3.8, 23.9 ± 5.1 and 23.1 ± 4.6) or cumulative CSD duration (292.6 ± 79.1, 297.1 ± 48.1 and 267.6 ± 73.9) (Additional files: Figures 1 and 2). Direct cortical cathodal stimulation with stepwise escalating intensities and durations triggered CSD in all rats.

**Table 2:** Effect of acute sleep deprivation on CSD electrical threshold

Group	n	CSD Threshold (μC)	
		*mean (range)*	*average ± SD*
Control	19	50 (1–800)	108 ± 227
6 h sleep deprivation	11	20 (1–400)	63 ± 116
12 h sleep deprivation	14	10 (1–300)*	41 ± 76*
6 h and 12 h sleep deprivation	25	15 (1–400)*	50 ± 93*

Sleep deprivation of 12 h but not 6 h decreased the electrical CSD threshold. All controls (6 h controls and 12 h controls) were pooled for statistical analysis, as no significant difference was found between the two control groups

*p < 0.01 vs. control

**Table 3:** Effect of sleep deprivation on CSD propagation speed and propagation failure

Group	n	Propagation speed (mm/min)	Propagation Failure (%)
Control	30	3.0 ± 0.4	31 ± 12
6 h sleep deprivation	17	3.0 ± 0.5	32 ± 13
12 h sleep deprivation	11	3.0 ± 0.3	30 ± 12

Acute sleep deprivation did not affect propagation speed or propagation failure of CSD in rats. Control groups for 6 h and 12 h sleep deprivation were pooled for statistical analysis, as no significant difference was found between the two control groups

Measurements were made of the first CSD recorded after topical KCl application

Values are average ± SD

### Chronic sleep deprivation did not affect CSD susceptibility

**Fig. 4:**
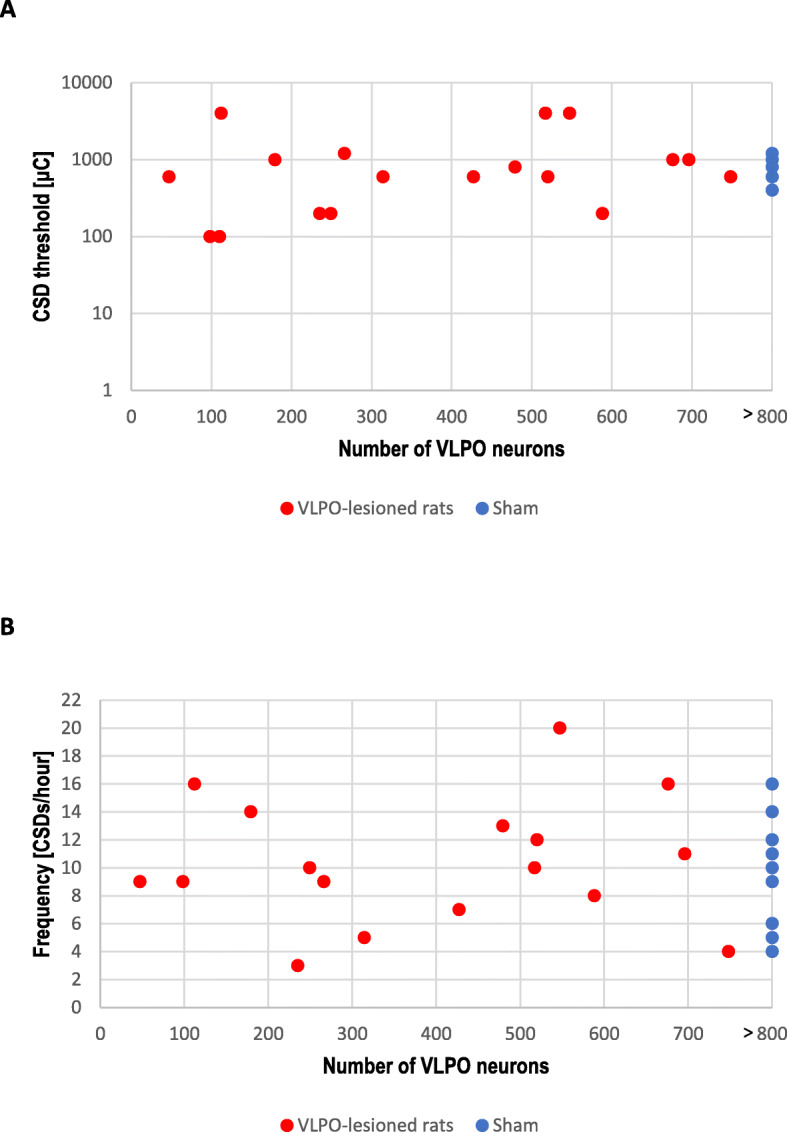
CSD threshold and frequency in VLPO-lesioned rats. Electrical CSD threshold (**a**) and CSD frequency upon topical continuous KCl (**b**) were not altered in rats after chronic sleep deprivation, 6 or 12 weeks after lesioning of the ventrolateral preoptic nucleus. The x-axis indicates the number of intact VLPO neurons after the lesioning procedure. The number of VLPO neurons for sham animals was set to > 800

Histological analysis showed that VLPO lesioning reduced the number of intact VLPO neurons, as detailed in Fig. 4. Continuous topical KCl application evoked repetitive CSDs in all rats. There was no difference between VLPO lesioned rats and controls, and there was no correlation between the number of intact VLPO neurons and CSD electrical threshold (1100 ± 1295 vs. 767 ± 320; Fig. 4a), CSD frequency (10.5 ± 4.3 vs. 9.7 ± 4.1; Fig. 4b) or for any other CSD parameter (propagation speed: 3.0 ± 0.5 vs. 3.3 ± 0.8; duration of first CSD: 23.8 ± 7.8 vs. 26.3 ± 7.8; cumulative CSDs duration: 174.2 ± 72.8 vs. 175.1 ± 44.7) (Additional files: Figure 3a and b).

## Discussion

We investigated the effect of sleep deprivation on CSD susceptibility, the electrophysiological correlate of migraine aura. Our experiments show that acute sleep deprivation for both 6 and 12 h, dose-dependently, increases CSD susceptibility in rats, as evidenced by a reduced electrical threshold for CSD induction in the 12 h sleep deprived group, and an increased frequency of CSD upon continuous topical application of KCl in both 12 h and 6 h sleep deprived groups. In order to maximize reliability and relevance of our findings, CSD susceptibility was tested using two well-established independent but complementary experimental paradigms (KCl application and electrical stimulation) that provided coherent results.

Clinically, sleep deprivation or excessive sleep, as well as other sleep disturbances are among to the most common attack triggers reported by patients with primary headaches (e.g. migraine without aura [25, 29], migraine with aura [30], familial hemiplegic migraine [31], tension-type headache [32, 33]). Conversely, sleep is associated with the resolution or relief of migraine attacks [34, 35]. Over half of reported migraine attacks are followed by daytime sleepiness and migraineurs often choose to sleep looking for relief from their headache [35]. Similarly, in animals, CSD is followed by an increase of NREM sleep phase duration, suggesting an increased need for sleep after an attack [36]. Patients with chronic migraine report shorter nightly sleep times than those with episodic migraine, and are more likely to exhibit difficulties falling asleep, staying asleep, are more prone to develop sleep triggered headache, and choose to sleep because of headache [35, 37]. The quality of sleep is decreased in adults with migraine [38, 39] and over half of them report difficulty initiating and maintaining sleep, at least occasionally [35]. Short sleepers, who routinely sleep 6 h or less per night, exhibit more severe headache patterns and are more likely to develop morning headaches on awakening than individuals who sleep longer [35]. Taken together, it appears there is a relationship between migraine and sleep disturbance, with a possible cause-effect relationship not being entirely clear at this point.

Consistent with these epidemiological findings, increasing evidence suggests that sleep deprivation has adverse effects on several pathways involved in headache, including cortical excitability, neurotransmission and neurogenesis [40]. Sleep disturbance impairs endogenous pain-inhibitory function and increases spontaneous pain, in particular headache [41]. Lack of sleep has been shown to increase cortical excitability, as a possible mechanism to explain our findings of an increased CSD susceptibility in sleep-deprived rats. For example, 4 h of sleep deprivation has been shown to enhance intrinsic cortical activity and excitability in rats, possibly through a Ca^2+^-dependent mechanism [42]. Similarly, in healthy humans using single pulse transcranial magnetic stimulation, it has been observed that sleep deprivation enhances cortical excitability with reduction of short intracortical inhibition [43]. As a possible underlying mechanism, sleep deprivation has been shown to increase levels of the excitatory neurotransmitter glutamate in rat cerebral cortex [44]. Moreover, sleep deprivation induces an increase in the expression of glutamate receptors in rat hippocampus, which is involved in increased susceptibility of short and long-term depression in that area [45]. Glutamatergic mechanisms have been implicated in CSD susceptibility [46], and, thus, an increase in glutamatergic neurotransmission could explain the sleep deprivation-induced increase in CSD susceptibility. In addition, lack of sleep may enhance CSD susceptibility by inhibiting the Na^+^/K^+^-ATPase in the setting of energy failure secondary to oxidative stress. The surge in adenosine triphosphate (ATP) levels, the energy currency of brain cells, occurs when neuronal activity is reduced, as during sleep. The levels of phosphorylated AMP-activated protein kinase (P-AMPK), important for cellular energy sensing and regulation, are lower during the sleep-induced ATP surge than during wake or sleep deprivation [47]. Accordingly, prolonged sleep deprivation significantly decreases the activity of anti-oxidative enzymes such as superoxide dismutase and glutathione peroxidase that regulate the level of reactive oxygen species [48] and has been shown to increase lipid peroxidation in the rat hippocampus, thalamus and hypothalamus, leading to a higher oxidative stress in these brain regions compared to others [49].

Another potential link between migraine, sleep deprivation and CSD is altered neurotransmitter dynamics. For example, adenosine has been implicated in migraine pathophysiology, because adenosine levels were shown to be elevated during migraine attacks and administration of adenosine can precipitate migraine attacks [15]. Moreover, the adenosine A2A receptor gene haplotype was found to be associated with migraine with aura [50]. Adenosine has been shown to be elevated after CSD in brain slices under conditions likely to trigger CSD in vivo, and adenosine receptor activation has been shown to be involved in the prolonged depression of synaptic transmission after CSD [51]. At the same time, a role for adenosine in sleep regulation has been suggested by studies showing a progressive increase in extracellular adenosine in the basal forebrain during prolonged wakefulness [52], possibly explaining the potential CSD-mediated negative effect of sleep deprivation on migraine.

The high prevalence of sleep disturbances in migraineurs could also depend in part on a dysregulation of the cyclic secretion of melatonin. During a migraine attack, the plasma levels of melatonin are decreased [53], and evidence suggests that administering melatonin to migraine sufferers relieves pain and decreases headache recurrence in some cases [54]. Accordingly, melatonin slows down retinal spreading depression [55] and can attenuate the process of trigeminovascular nociception induced by CSD in rats [56]. In addition, serotoninergic processes have been implicated in mediating the migraine promoting effect of sleep deprivation. Serotonin is a neuromodulator with a pivotal role in regulating several central nervous system activities, including pain threshold and sleep induction. Sleep deprivation causes an enhancement of serotonergic neurotransmission in the brain, as suggested in animal studies. In sleep-deprived rats, elevated serotonin levels have been measured in the hippocampus [57] and in serotonergic raphe nuclei [58], which, with their widespread cortical projections, are part of the monoaminergic wake promoting system. Accordingly, human in vivo studies showed that plasma levels of serotonin and its precursor tryptophan exhibit a significant increase during sleep deprivation compared to sleep [59] and that a single night of total sleep deprivation causes significant increases of serotonin 2A receptor binding potentials in a variety of cortical regions [60]. Future experiments could aim to clarify the role of melatonin and serotonin in mediating the effect of sleep deprivation on CSD, for example by testing if melatonin supplementation or anti-serotonergic drugs diminish or abrogate the sleep deprivation-induced increase in CSD susceptibility.

Recently, Kilic et al. reported that the enhanced susceptibility to CSD after sleep deprivation may be due to impaired K+ and glutamate clearance by astrocytes consequent to sleep deprivation-induced suppression of glycogen use and synaptic energy substrate deficiency [61]. The authors demonstrated that sleep deprivation for 6 h using the same “gentle handling method” decreased threshold for CSD induction and prolonged the duration of CSDs without changing the CSD propagation speed and frequency, which, on the contrary, had increased in our experiments. Furthermore, this study reported that the decreased CSD thresholds could be recovered via the use of an alternate energy supply (D-glucose or L-lactate superfusion), linking decreased CSD thresholds to an activity/energy mismatch resulting from prolonged wakefulness [61]. An alternate energy supply was able to revert the enhancement of CSD susceptibility and reduced clearance of K+ during synaptic activity. Differences between results of the Kilic study and ours could be attributed to species differences, because mice show a predominantly vasoconstrictive response to CSD, which is in contrast to the biphasic vascular response that is seen in humans or rats, used in our experiments. In addition, Kilic et al. administered urethane/xylazine as anesthetic, which has a different effect on cortical excitability than isoflurane, which was used in our study [62].

We also tested the effect of chronic sleep deprivation on CSD susceptibility in rats sustaining the loss of VLPO neurons, a previously validated model of chronic sleep restriction [26]. It is well established that the VLPO is a key sleep-promoting cell group in the hypothalamus, and, already in 1930, von Economo reported that lesions of this region cause prolonged insomnia in humans [63]. Neurons in the preoptic hypothalamus are important regulators of sleep onset and sleep maintenance [64]. VLPO is thought to play a major role in causing sleep by GABAA receptor-mediated hyperpolarization and inhibition of histaminergic neurons in the tuberomammillary nucleus [65], which are important for promoting wakefulness [66]. VLPO lesions induce long-lasting insomnia in rats, with a 60–70% decrease in delta power and a 50–60% decrease in NREM sleep time [26]. VLPO lesion experiments allowed us to study the effect of chronic partial sleep loss on CSD without continual stressful experimental interventions, to avoid a possible confounding impact of stress on CSD. We here show that CSD susceptibility is not altered 6 weeks or 12 weeks after VLPO lesioning, suggesting that the effect of acute sleep deprivation on CSD is not mediated via reduced NREM sleep, that *acute total* sleep deprivation has a different effect on cortical excitability than *chronic partial* sleep restriction, and/or that compensatory mechanisms cause adaptation of cortical excitability within the *chronic* setting.

Our study has several limitations, including the fact that animal sleep deprivation models may not allow direct extrapolation to patients. However, gentle handling is very effective at inducing total sleep deprivation as determined by electroencephalography [67] and seems to be a valid model of typical sleep deprivation in humans. While the gentle handling method is less stressful than other methods that induce sleep deprivation, it still may be a cause of stress. It is therefore possible that the pathways induced by sleep deprivation in our animal model are different from those that occur in humans who consciously decide to stay awake or delay sleep. However, we feel that acute stress may not be important for our study endpoint, CSD susceptibility, as acute stress has recently been shown to not affect CSD susceptibility in rodents [68]. In addition, we used an invasive method to induce and record CSD, which may have affected our endpoint of CSD susceptibility. However, assessment of CSD susceptibility with electrocortical recordings, and CSD induction with KCl and electrical stimuli are the most established methods, used in many other respected studies in the field [17–19]. Another possible shortcoming is the fact that rats were anesthetized during CSD assessment, and it has been shown that anesthetics modify CSD threshold [62]. Future experiments could perform CSD recordings in the sleep-deprived awake non-anesthetized rat, using non-invasive techniques for CSD induction, such as optogenetics [69]. In addition, assessment of additional migraine surrogates might further underscore the relevance of our findings for migraine, such as monitoring of face grimacing [70], and measurement of blood levels of adenosine, melatonin or serotonin - factors that are important in migraine and might mediate the effect of sleep deprivation on CSD. Finally, future experiments that confirm our results in mutant mice carrying human migraine mutations, such as FHM, and/or in female rodents, could further underscore the clinical relevance of our findings.

## Conclusions

Primary headaches and sleeping problems are highly prevalent and cause considerable social and familial challenges. Sleep disorders and migraine seem to share pathophysiological pathways with a bidirectional influence, making it difficult to discern cause and effect. This study shows that acute sleep deprivation enhances CSD susceptibility, similar to other migraine promoting factors such as female gonadal hormones or genetic mutations. Acute sleep deprivation increases CSD frequency during topical KCl application and decreases the electrical threshold of CSD upon electrical stimulation. Furthermore, our results show that partial *chronic* sleep restriction/fragmentation by VLPO-lesioning, with predominant loss of NREM sleep, does not affect CSD susceptibility. These findings further underscore the importance of CSD as a migraine substrate, and open up new potential therapeutic avenues, suggesting that headache management should identify and treat associated sleep disorders.

## Supplementary information

**Additional file 1 : Figure 1.** Acute sleep deprivation: Duration of first CSD. Duration of the first CSD was not reduced after 12 h or 6 h of sleep deprivation. Each circle represents the results of an individual rat. All controls (6 h controls and 12 h controls) were pooled for statistical analysis, as no significant difference was found between the two control groups. **Figure 2.** Acute sleep deprivation: Cumulative duration of CSDs. Cumulative duration of CSDs was not reduced after 12 h or 6 h of sleep deprivation. Each circle represents the results of an individual rat. All controls (6 h controls and 12 h controls) were pooled for statistical analysis, as no significant difference was found between the two control groups. **Figure 3.** Duration of first CSD and cumulative duration of CSDs in VLPO-lesioned rats. Duration of first CSD (**A**) and cumulative duration of CSDs upon topical continuous KCl (**B**) were not altered in rats after chronic sleep deprivation, 6 or 12 weeks after lesioning of the ventrolateral preoptic nucleus. The x-axis indicates the number of intact VLPO neurons after the lesioning procedure. Each circle represents the results of an individual rat. The number of VLPO neurons for sham animals was set to 800.

## Data Availability

Dataset available from the corresponding author on reasonable request.

## Abbreviations

ATP: Adenosine triphosphate
CADASIL: Cerebral autosomal dominant arteriopathy with subcortical infarcts and leukoencephalopathy
CSD: Cortical spreading depolarization
DC: Extracellular steady
NREM: Nonrapid-eye movement
REM: Rapid-eye movement
VLPO: Ventrolateral preoptic nucleus
