# Supplementary material for: Acute sleep deprivation enhances susceptibility to the migraine substrate cortical spreading depolarization
Source: J Headache Pain. 2020 Jul 6;21(1):86. doi: 10.1186/s10194-020-01155-w (PMC7339460; doi:10.1186/s10194-020-01155-w)
Supplement: Supplementary file 1 — Additional file 1 : Figure 1. Acute sleep deprivation: Duration of first CSD. Duration of the first CSD was not reduced after 12 h or 6 h of sleep deprivation. Each circle represents the results of an individual rat. All controls (6 h controls and 12 h controls) were pooled for statistical analysis, as no significant difference was found between the two control groups. Figure 2. Acute sleep deprivation: Cumulative duration of CSDs. Cumulative duration of CSDs was not reduced after 12 h or 6 h of sleep deprivation. Each circle represents the results of an individual rat. All controls (6 h controls and 12 h controls) were pooled for statistical analysis, as no significant difference was found between the two control groups. Figure 3. Duration of first CSD and cumulative duration of CSDs in VLPO-lesioned rats. Duration of first CSD (A) and cumulative duration of CSDs upon topical continuous KCl (B) were not altered in rats after chronic sleep deprivation, 6 or 12 weeks after lesioning of the ventrolateral preoptic nucleus. The x-axis indicates the number of intact VLPO neurons after the lesioning procedure. Each circle represents the results of an individual rat. The number of VLPO neurons for sham animals was set to 800. [file 10194_2020_1155_MOESM1_ESM.docx]

Figure 1. Acute sleep deprivation: Duration of the first CSD.

Duration of the first CSD was not reduced after 12 hours or 6 hours of sleep deprivation. Each circle represents the results of an individual rat.

All controls (6h controls and 12h controls) were pooled for statistical analysis, as no significant difference was found between the two control groups.

Figure 2. Acute sleep deprivation: Cumulative duration of CSDs.

Cumulative duration of CSDs was not reduced after 12 hours or 6 hours of sleep deprivation. Each circle represents the results of an individual rat.

All controls (6h controls and 12h controls) were pooled for statistical analysis, as no significant difference was found between the two control groups.

Figure 3. Duration of first CSD and cumulative duration of CSDs in VLPO-lesioned rats.

**A**

**B**

Duration of first CSD (**A**) and cumulative duration of CSDs upon topical continuous KCl

(**B**) were not altered in rats after chronic sleep deprivation, 6 or 12 weeks after lesioning

of the ventrolateral preoptic nucleus. The x-axis indicates the number of intact VLPO

neurons after the lesioning procedure. Each circle represents the results of an individual

rat. The number of VLPO neurons for sham animals was set to 800.
